# COVID-19 and psychiatric education: From postgraduate to continuous medical education

**DOI:** 10.1192/j.eurpsy.2021.141

**Published:** 2021-08-13

**Authors:** M. Casanova Dias

**Affiliations:** National Centre For Mental Health, School Of Medicine, Cardiff University, Cardiff, United Kingdom

**Keywords:** Continuous Medical Education, COVID-19, postgraduate psychiatry training, psychiatric education

## Abstract

COVID-19 has impacted psychiatric education at many levels from postgraduate training to Continuous Medical Education (CME). We invited participants at the European Union of Medical Specialists (UEMS) who are national representatives to share how COVID-19 has impacted postgraduate training and Continuous Professional Development (CPD) in their countries. They were asked to report the challenges but also the opportunities created by the pandemic and their answers were analysed. Several themes emerged. Challenges in postgraduate training have been absences caused by COVID, redeployment, reduced interactions and postponement of assessments. The mental health of trainees was affected, including burnout. Interestingly in some places, like Denmark, training was less impacted as psychiatry was designated as ‘critical’ and therefore no redeployment. Exams have moved online and there have been concerns about cheating in the new format. In countries where it is obligatory to be uptodate with CME/CPD to maintain medical registration, the usual requirements were waived. Conferences and live events have moved online and webinars became popular and widely accepted. Some positive developments included rapid adoption of technology, for consultations and training, increased relevance of CPD/CME, emphasis on team cohesion and recognition of the need for self-care and team support. The pandemic also fostered international collaboration, e.g. sharing guidelines for new ways of working. Some of the innovations described, mainly related to the adoption of technology and remote working will likely be taken in the future. However, the sentiment remains that live exchanges are valuable and should be resumed as soon as it is safe.

**Disclosure:**

No significant relationships.

